# Designing an Image Encryption Scheme Based on Compressive Sensing and Non-Uniform Quantization for Wireless Visual Sensor Networks

**DOI:** 10.3390/s19143081

**Published:** 2019-07-12

**Authors:** Qian Shen, Wenbo Liu, Yi Lin, Yongjun Zhu

**Affiliations:** 1 College of Automation Engineering, Nanjing University of Aeronautics & Astronautics, Nanjing 210000, China; 2 School of Electronic & Information Engineering, Suzhou University of Science and Technology, Suzhou 215009, China

**Keywords:** chaos, image encryption, compressive sensing, wireless visual sensor networks

## Abstract

Wireless visual sensor networks (WVSN) have been widely used to capture images in the fields of monitoring, intelligent transportation, and reconnaissance in recent years. Because of the wireless transmission mode and the huge amount of image data, major challenges in this application are frequent information stealing, big data problems, and harsh communication circumstances. Some encryption schemes based on compressive sensing (CS) and chaotic systems have been proposed to cope with these threats, but most of them are vulnerable against the chosen-plaintext attack (CPA). To remedy these defects, this paper designs a novel method based on non-uniform quantization (NQ). Then, in order to evaluate the true compression ratio (CR), our work takes into account limited data precision in cipher images, while most papers ignored this fact and calculated CR with the assumption of infinite data precision. Besides, to eliminate the periodic windows in the bifurcation diagram of the logistic map (LM), an optimized logistic map (OLM) is designed. Furthermore, simulation results prove that the performance of anti-jamming in the proposed cryptosystem is better than that in existing schemes under the condition of strong noise interference or severe data loss. In conclusion, the proposed method could improve the performance of security and anti-jamming for WVSN.

## 1. Introduction

The structure of conventional data compression methods for wireless visual sensor networks (WVSN) could be divided into two parts: the acquisition and the reduction [1]. This means some redundant information must be captured before being removed, which leads to an unnecessary expense. Since a meaningful image always has a sparse representation under some basis, we might abandon the compression process by means of simply acquiring the useful information. Therefore, a promising technique called compressive sensing (CS) [2] was proposed in 2006, and it was soon widely used for image compression in WVSN owing to its advantage of consumption reduction at sensor nodes [3].

At present, the security of WVSN has been challenged by modified cracking methods [4]. As a result, cryptosystems should be upgraded. Fortunately, chaotic systems offer good ideas to solve this problem due to their characteristics of deterministic randomness, initial value sensitivity, boundedness, etc. [5,6]. The most common way is encrypting images with measurement matrices generated by chaotic systems [7,8]. For instance, Huang’s work [9] designed a Toeplitz matrix whose elements were produced by a two-dimensional generalized Arnold map, and then, it was employed to conduct the measurement operation. The work in [10] also embedded a tent map into CS in a similar way. However, these methods were proven to be vulnerable against the chosen-plaintext attack (CPA) [11]. Therefore, a great number of studies continued to seek further improvements based on this framework. Some researchers focused on expanding the space of parameter values, but their schemes of combining multiple chaotic systems [12] or modifying existing chaotic maps [13,14,15] mostly had a few flaws of massive period windows or low Lyapunov exponents. More recently, CS and chaos have been mixed to build a complex structure to obtain extra security assurance. For example, Zhou et al. [16] created a serial and parallel hybrid scheme, which was beneficial to apply mature encryption algorithms based on chaos. This measure indeed helped to enhance the security performance, but it was still vulnerable against CPA because the measurement process could be considered as a fixed linear projection. In fact, if a cryptosystem can resist CPA, it could be considered secure under all kinds of attacks like the chosen-ciphertext attack (CCA), etc. [17]. Some recent algorithms based on chaos might tackle this problem, but they are inappropriate for wireless channels because of their poor robustness or high complexity [6,18,19,20]. For example, the scheme based on the orbit variation of the phase diagram [6] could indeed resist CPA owing to the relevance between the encryption sequence and plaintext. However, the structure of cipher block chaining (CBC) would cause low performance in anti-jamming, which determines that the cryptosystem can only be used for lossless application. As a result, this article proposes a novel encryption scheme with desirable anti-interference performance based on CS and non-uniform quantization (NQ), which also achieves resisting CPA with less consumption [6,21,22].

On account of the massive data acquired by sensor networks, another major issue in WVSN is the efficiency of communication [23,24,25]. Zhang’s work [7] claimed that its scheme could encrypt data and compress data simultaneously, but it neglected the fact that the space usage for each element in cipher data with no quantization was more than that in plain data. Therefore, the result of the compression ratio (CR) in [7] was not accurate. To get a more precise result, we take into account the effect of data precision. Then, the formula of CR could be optimized from:

(1) $$cr=\frac{L_{C}}{L_{P}}\times 100\%$$

to:

(2) $$cr=\frac{L_{C}\times DP_{C}}{L_{P}\times DP_{P}}\times 100\%,$$

where $L_{C}$ and $L_{P}$ are the number of pixels in a cipher image and the number of pixels in a plain image, respectively. Besides, $DP_{C}$ is the number of binary bits for each pixel in a cipher image, and $DP_{P}$ is the number of binary bits for each pixel in a plain image. For example, $L_{P}$ and $DP_{P}$ of an eight-bit gray-scale image of size 512×512 are 262,144 and eight, respectively. As analyzed above, using CS alone is not enough to improve the efficiency in transmission. Therefore, the measurement result of a scene is usually quantized to be a sequence with finite data precision. To be specific, the size of data in a cipher image should be smaller than that in a plain image. The conventional method to solve this problem is applying uniform quantization (UQ) to the measurement result. However, the accuracy loss could not be neglected due to its irrational allocation of quantization resources. The quantization error in the centrally-distributed region would be large if consuming too much quantization resources in dealing with few values at the edge of the distribution region for measurement results, according to the characteristic that the pseudo-random measurement results are close to a Gaussian distribution [26]. Since the importance of each element in the measurement results is equivalent, our idea is to design a self-adaptive NQ method to concentrate quantization resources in the centralized area of data. The performance of this scheme will be evaluated in Section 4.2.

In conclusion, the main contributions of this work contain four aspects. First, the optimized logistic map (OLM) was designed to expand the parameter value space and eliminate the period windows in current chaotic systems. Second, our work modified the formula of CR by taking into account the limited data precision in cipher images, while most papers ignored this fact and calculated CR with the assumption of infinite data precision. Third, in order to maintain the ability of anti-interference while ensuring that the encryption framework based on CS can resist CPA, a self-adaptive NQ method was proposed. Ultimately, the performance of anti-jamming in the proposed cryptosystem was better than that in existing schemes according to the theories’ analysis and simulation results.

The rest of this paper is organized as follows. The preliminary knowledge and designed OLM are introduced in the next section. Then, in Section 3, the framework and detailed steps of the proposed cryptosystem are illustrated. After that, simulation results and the corresponding theories’ analysis are described in detail in Section 4. The last section concludes all work mentioned in this paper.

## 2. Preliminary Knowledge and Designed OLM

This section briefly reviews the basic knowledge of CS [2] and chaos. Besides, OLM is proposed to ensure stable chaos within the whole range of parameters.

### 2.1. Compressive Sensing

Assume that one signal $x\in R^{n}$ could be represented as a sparse form under basis $\Psi \in R^{n\times n}$, and it is expressed with x=Ψθ [27]. Then, with a proper measurement matrix, the sampling process in CS could be described as:

(3) y=Φx,

where Φ is an m×n measurement matrix with m<n, and it is applied to extract information from an *n*-dimensional signal x. Besides, $y\in R^{m}$ stands for the measurement result, which contains all information about the original signal. Obviously, it is hard to reconstruct x in Equation (3) due to the reason that the dimension of y is less than x.

However, this function could be further extended to:

y=Φx=ΦΨθ=Aθ,

where A is the result of Φ times Ψ. Theory analysis [28] has proven that the original signal x could be recovered accurately with:

(4) $$x=\underset{x}{\arg\min}\;{∥\theta ∥}_{0},\;\;s.t.\;\;A\theta =y$$

if A obeys the restricted isometry property (RIP). Here, ${∥\theta ∥}_{0}$ stands for the $l_{0}$-norm of vector θ. The RIP ensures that different signals with the same sparsity can be distinguished. Actually, Equation (4) is an NP-hard problem, which could be solved by greedy algorithms such as orthogonal matching pursuit (OMP), regularized orthogonal matching pursuit (ROMP), etc. Furthermore, under certain conditions, Equation (4) could be relaxed as another problem [29]:

$$x=\underset{x}{\arg\min}\;{∥\theta ∥}_{1},\;\;s.t.\;\;A\theta =y,$$

where ${∥\theta ∥}_{1}$ is the $l_{1}$-norm of vector θ [30]. Many methods have been proposed to solve this convex optimization problem, such as interior point methods, gradient projection methods, etc.

### 2.2. Proposed OLM

A chaotic sequence refers to the iteration values generated by the random motion of a deterministic dynamic system that is sensitive to initial values. As we know, the attributes of deterministic randomness, boundedness, parameters, initial value sensitivity, etc., are appropriate to apply in encryption.

**Figure 1:**
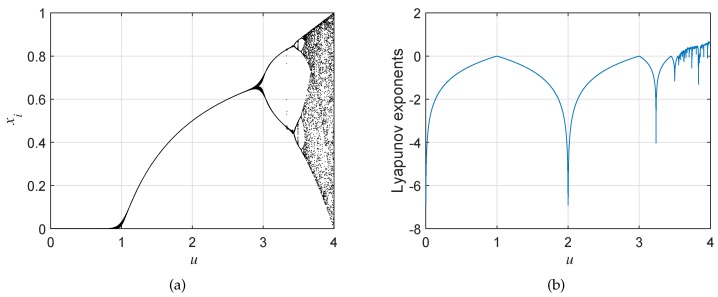
The (**a**) bifurcation diagram and (**b**) Lyapunov exponents of LM.

The logistic map (LM) is a classic map that has been widely used in the generation of chaotic sequences. Its mathematical model is:

$$x_{i+1}=u\cdot x_{i}\cdot \left(1-x_{i}\right),$$

where parameter *u* ranges from 0–4. As shown in Figure 1a, LM is likely to be chaotic if u∈[3.57,4]. However, there exist some periodic windows within this range according to Lyapunov exponents, demonstrated in Figure 1b. This means that, even if parameter *u* meets the requirement, the iteration values generated by LM might be periodic, which is adverse to the application in cryptosystems.

**Figure 2:**
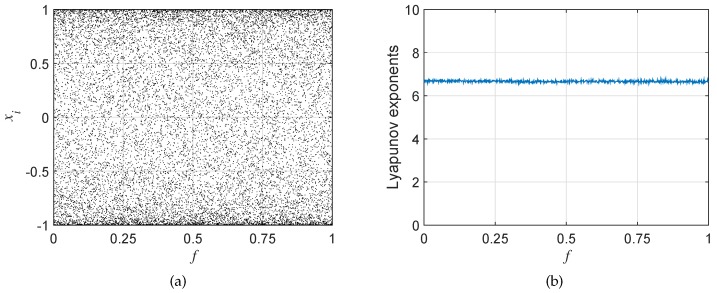
The (**a**) bifurcation diagram and (**b**) Lyapunov exponents of OLM.

This paper modified LM with trigonometric functions to eliminate its defect. The optimized map called OLM is defined as:

(5) $$x_{i+1}=\sin\left(\left(f+500\right)\cdot x_{i}\cdot \left(1-x_{i}\right)\cdot \pi \right)\cdot \cos\left(\left(f+500\right)\cdot x_{i}\cdot \left(1-x_{i}\right)\cdot \pi \right)\cdot 2,$$

where *f* is a control parameter. According to Figure 2b, OLM would be chaotic as long as f∈[0,1]. Besides, its bifurcation diagram drawn in Figure 2a shows that iteration values are randomly distributed in [-1,1]. Obviously, the chaotic sequence generated by OLM is more appropriate for encryption applications.

## 3. The Proposed Cryptosystem

**Figure 3:**
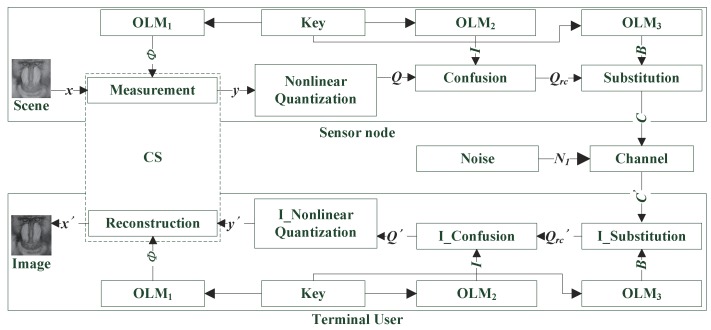
The framework of the proposed system.

In some recent studies about WVSN, the traditional charge coupled device (CCD) camera was replaced by a single pixel camera [31], which contributes to consumption reduction in sensor nodes. Based on this, the framework proposed by this paper was designed as Figure 3 shows. A chaotic sequence controlled by the secret key, as can be seen, was utilized to generate measurement matrix Φ for CS. After the measurement result y was obtained, an operation of NQ based on a parameter-variable non-linear transform was executed to get quantized data Q. Then, another two chaotic sequences were utilized to confuse and substitute Q separately, which helped to improve the security of cipher data C. In the decryption process, symbol “I_” stands for an inverse operation. According to this scheme, the decryption part was simply an inverse operation of encryption one mentioned above, except for a reconstruction step. As a result, only the encryption process is elaborated in this section.

**Figure 4:**
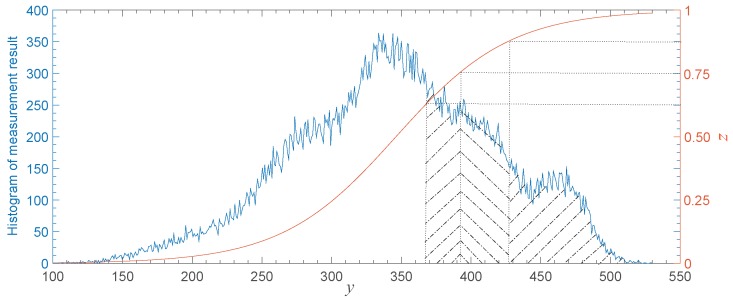
The process of NQ.

The encryption process could be divided into three steps:

1. Measurement:
  In the proposed scheme, the scene at a sensor node was directly measured by a single pixel camera whose micro-mirror array was controlled by a measurement matrix. Generally speaking, in order to satisfy RIP, most measurement matrices are the Gaussian random matrix and the Bernoulli random matrix. In WVSN with security requirements, the measurement matrix is usually generated by a secret key that should be transmitted; hence, a random matrix is no longer suitable. In 2012, Chen et al. [32] proved that matrices constructed by chaotic sequences perform better. More importantly, this kind of matrix could be generated by few parameters, which helps to shrink the size of the key conspicuously. Furthermore, the Toeplitz-structured chaotic measurement matrix has been verified to be capable of satisfying RIP with high probability [33]. To further shorten the length of the chaotic sequence required, a cyclic-structured chaotic matrix:
  $$C=\left(\begin{array}{cccc} t_{n} & t_{n-1} & \cdots & t_{1}\\ t_{1} & t_{n} & \cdots & t_{2}\\ \vdots & \vdots & \ddots & \vdots \\ t_{m-1} & t_{m-2} & \cdots & t_{m} \end{array}\right)$$
  was employed to construct Φ, whose elements $t_{1}$, $t_{2}$, ⋯, $t_{n}$ are a normalized chaotic sequence generated by OLM${}_{1}$.
2. Quantization:
  The quantization process could be divided into two parts: the parameter-variable non-linear transform and the conventional UQ. First, the measurement result y was transformed by a non-linear function:
  (6) $$z=\frac{1}{1+e^{-a(y-oset)}},$$
  where *a* and oset are the convergence rate and convergence center of this function, respectively. Now that the elements in the measurement matrix are pseudo-random, the histogram of measured data is similar to a Gaussian distribution according to the central limit theorem and the Berry–Esseen theorem [26]. As shown in Figure 4, the elements of y tended to concentrate near the center. Therefore, with appropriate parameter values, the result of the non-linear transform by Equation (6) could be approximately uniformly distributed, which is suitable to make good use of quantization resources. The value of oset in Equation (6) was set to the average of elements in y, which is marked with $y_{mean}$. It determines the displacement of the function. Besides, the standard deviation of y marked with δ was utilized to calculate parameter *a*, which controls the convergence rate of the functional curve. To be specific, the values 0.05 and 0.95 on the right axis of Figure 4 correspond to values $y_{mean}-2\delta$ and $y_{mean}+2\delta$ on the horizontal axis, which ensures 95% of quantization resources could deal with most elements in *y*. Hence, *a* could be calculated with:
  (7) $$a=\frac{\ln\left(1/0.05-1\right)}{2\delta }$$
  according to Equation (6). In this way, the parameters of the non-linear transform function could adapt to different measurement results. For the convenience of intuitive understanding, several quantitative series are marked with oblique lines in Figure 4. Obviously, the number of elements in different oblique lines would be similar. After the non-linear transformation was completed, classical UQ methods could be directly utilized to convert the transformed result z to be the quantized value Q.
3. Confusion and substitution:
  After that, the process of confusion and substitution [34,35] was appended to increase the security of quantized data. In the confusion process, OLM${}_{2}$ was firstly applied to iterate *t* times to obtain a chaotic sequence, whose length was the same as Q’s. Then, we sorted this sequence by the values to acquire an index sequence I ranging from 1–*t*. Afterwards, we reordered the elements in Q by sequence I to get confused sequence $Q_{\mathrm{rc}}$. In the substitution step, the CBC structure was avoided to enhance the anti-interference performance of the proposed system. Chaotic sequence B was utilized to conceal the information by:
  (8) $$C\left(i\right)=Q_{rc}\left(i\right)⊕B\left(i\right),\;\;i\in \left\{1,2,...,t\right\},$$
  where C is the cipher image to be sent and *i* represents the index of the vectorized cipher image.

Eventually, cipher image C would be sent to the terminal user directly or through some medium nodes. Additive noise $N_{1}$ was drawn in the framework to model the interference in the wireless channel.

## 4. Simulation and Analysis

Although the scenarios at sensor nodes could be converted to plain images of various in sizes, gray-scale levels, and color patterns, they could all be separated easily [6]. For simplicity, this paper assumed that all the plain and cipher images were eight-bit gray-scale. That is to say, all pixel values in this paper were unsigned integers in eight bits (UINT8) ranging from 0–255. Studies have proven that information of a meaningful image always has a sparse representation under some basis. Without loss of generality, the discrete cosine transformation (DCT) matrix was taken as the sparse basis Ψ. Then, we assumed a vector $x\in R^{n}$ was the vectorized scene of size $\sqrt{n}\times \sqrt{n}$ at a sensor node, and it could be recovered precisely if measurement matrix Φ met the requirements proposed in Section 2.1. The cyclic-structured chaotic matrix mentioned above was utilized to construct measurement matrix Φ. Since this work does not focus on the research of reconstruction, a commonly-used greedy algorithm called OMP was directly applied in the reconstruction process. In this section, some standard images are taken as examples to evaluate the performance of the security and anti-jamming of the proposed scheme.

### 4.1. Security Performance

#### 4.1.1. Ability of Resisting CPA

Most cryptosystems could be simplified to:

(9) $$C_{w(i)}=f\left(P_{i},C_{w(i)-1},B_{w(i)}\right),$$

where w(i) is the element’s index in the vectorized cipher image associated with the $i^{\mathrm{th}}$ element in the vectorized plain image by the confusion operation. Besides, $C_{w(i)}$, $P_{i}$, $B_{w(i)}$, and f(·) are the $w{\left(i\right)}^{\mathrm{th}}$ element in the vectorized cipher image, the $i^{\mathrm{th}}$ element in the vectorized plain image, the $w{\left(i\right)}^{\mathrm{th}}$ element in the chaotic encryption sequence, and a fixed function containing exclusive or (XOR) and modulus operations, respectively. For a certain secret key, $B_{w(i)}$ could be considered as an invariant, which causes the result that $P_{i}$ only depends on $C_{w(i)}$ and $C_{w(i)-1}$. Obviously, it could be cracked easily by CCA [36] or CPA [21].

On the basis of the theories analyzed in [6], the most popular way to resist CPA and CCA is modifying the encryption sequence with the plain image as:

(10) $$C_{w(i)}=f\left(P_{i},C_{w(i)-1},h\left(B_{w(i)},P_{w^{-1}\left(w\left(i\right)-1\right)}\right)\right),$$

where h(·) is a fixed non-linear function. According to this model, a tiny residual in the recovered image would cause a huge error in the generation of the encryption sequence, which greatly affects the anti-jamming performance of a cryptosystem.

Fortunately, CS could solve this problem ideally. The mathematical model of existing schemes based on CS is:

(11) $$C_{w(i)}=f\left(\sum_{r=1}^{n}\Phi_{i,r}\cdot P_{r},B_{w(i)}\right),$$

where $\Phi_{i,r}$ represents the $i^{\mathrm{th}}$ row and the $r^{\mathrm{th}}$ column element in measurement matrix Φ and $P_{r}$ stands for the $r^{\mathrm{th}}$ element in the vectorized plain image. In Equation (11), each pixel $P_{r}$ in the vectorized plain image is influenced by all pixels in the corresponding cipher image, and each pixel in a cipher image is influenced by all pixels in the corresponding plain image. These characteristics could help to resist most cracking methods. For example, the work in [36] obtained the starting point for CCA by analyzing matrix T, which revealed the causality relations between cipher pixels and plain pixels in the decryption process. However, all elements in matrix T would be “1” for methods based on CS, which causes a failure in pursuing breakthroughs with T. Furthermore, due to the fact that each element in the cipher image involves all information about the plain image, the anti-jamming performance of the cryptosystem based on Equation (11) is better than those based on Equation (10).

Nevertheless, this scheme could be cracked within limited steps by some differential cryptanalysis methods based on CPA [21]. For example, with some plain images for which all elements are zeros, but only one element has a value of one, a cryptanalyst could acquire information about every column of measurement matrix Φ. This information could be accumulated and decomposed easily because of the linear relationship between the cipher image and the plain image. In conclusion, cryptosystems based on Equation (11) are vulnerable against CPA.

In the proposed scheme, the encryption process has been modified to:

(12) $$C_{w(i)}=f\left(g\left(\sum_{r=1}^{n}\Phi_{i,r}\cdot P_{r}\right),B_{w(i)}\right),$$

where g(·) stands for a parameter-variable non-linear function, whose parameters are relevant to the statistical characteristics of plain image P. In other words, this scheme would alter the encryption model as soon as the plain image changes. Moreover, it converts the relationship between cipher images and plain images from linear to non-linear, which helps to resist differential cryptanalysis methods based on CPA.

#### 4.1.2. Key Space and Key Sensitivity

The key space of a cryptosystem should be larger than $2^{112}$ to resist a brute-force attack [37]. In fact, this requirement is quite easy to be met by using various kinds of chaotic systems. For example, the parameter value *f* and initial value $x_{0}$ in an OLM could be taken as a secret key. Assume that the data type of the chaotic system is Float32 (32 binary bits); one OLM would require a key containing $2^{64}$ bits. Hence, the three chaotic maps applied in Figure 3 needed a total of $2^{192}$ bits for secret key, which was big enough to meet the requirement of key space. Furthermore, the length of a key could be adjusted according to the practical situations by means of locking some parameters or replacing OLM with a more complicated chaotic system.

**Figure 5:**
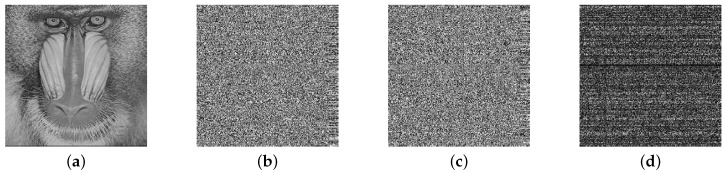
Key sensitivity in the encryption part. (**a**) Plain image of a baboon; (**b**) cipher image by the correct secret key; (**c**) cipher image by the changed secret key; (**d**) information variation between two cipher images.

**Figure 6:**
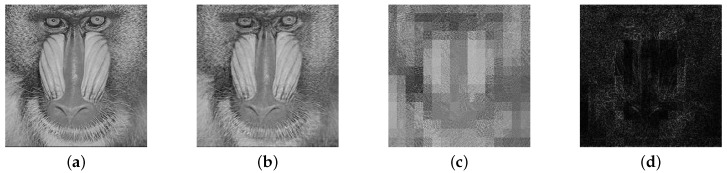
Key sensitivity in the decryption part. (**a**) Plain image of a baboon; (**b**) decrypted image with correct secret key; (**c**) decrypted image with wrong secret key; (**d**) difference between two decrypted images.

The key sensitivity of a cryptosystem requires that the cipher images encrypted with different secret keys should be different and that the decrypted images should be unrecognized with incorrect keys. This paper assumes that a tiny change of 0.001 in the initial value of OLM${}_{2}$ has occurred. Then, the corresponding cipher image would be drastically changed as shown in Figure 5. On the other hand, decryption is proven to fail in Figure 6 with the hypothesis that the parameter value in OLM${}_{2}$ contains an error of 0.001. Therefore, for our scheme, images can only be reconstructed by correct secret keys.

#### 4.1.3. Statistical Histogram

**Figure 7:**
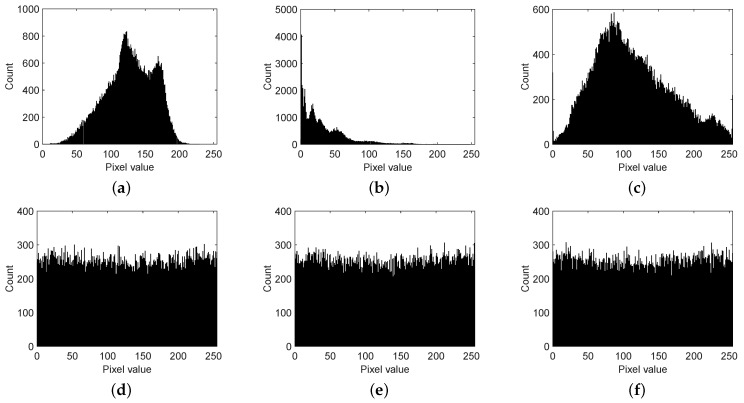
(**a**) Histogram of a plain image of a baboon. (**b**) Histogram of a plain image of a couple. (**c**) Histogram of a plain image of a bridge. (**d**) Histogram of a cipher image of a baboon. (**e**) Histogram of a cipher image of a couple. (**f**) Histogram of a cipher image of a bridge.

The statistical histograms of images could cause information disclosure to attackers, so cipher images should conceal this kind of information. As shown in Figure 7, all encrypted images were alternated to be uniformly distributed by the substitution method shown in Equation (8), and this was based on the pseudo-random sequence generated by OLM${}_{3}$.

#### 4.1.4. Correlation Coefficients

**Figure 8:**
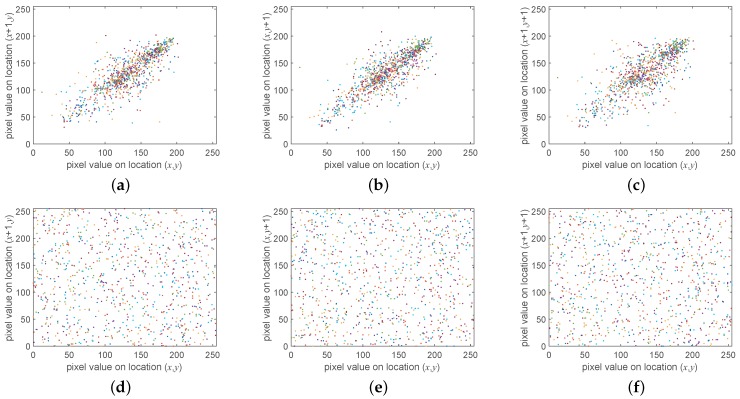
Correlation of adjacent pixel pairs for the baboon image. (**a**) Plain image by horizontal direction; (**b**) plain image by vertical direction; (**c**) plain image by diagonal direction; (**d**) cipher image by horizontal direction; (**e**) cipher image by vertical direction; (**f**) cipher image by diagonal direction.

Between two adjacent elements of a plain image, there always exists a strong correlation that is adverse to the security of the cryptosystem. As we know, the confusion process could eliminate this defect by randomly scrambling all elements of a image. To testify to the effect, the correlation of 4096 pairs selected by adjacent pixels in the vertical, horizontal, and diagonal directions, respectively, from the sample image of a baboon is drawn in Figure 8. Furthermore, the corresponding coefficients were calculated by the formula [34]:

(13) $$r_{xy}=cov\left(x,y\right)/\left(\sqrt{D(x)}\sqrt{D(y)}\right),$$

where:

$$cov\left(x,y\right)=1/N\cdot \sum_{i=1}^{N}\left(x_{i}-E\left(x\right)\right)\left(y_{i}-E\left(y\right)\right),$$

$$E\left(x\right)=1/N\cdot \sum_{i=1}^{N}x_{i},$$

$$E\left(y\right)=1/N\cdot \sum_{i=1}^{N}y_{i},$$

$$D\left(x\right)=1/N\cdot \sum_{i=1}^{N}{\left(x_{i}-E\left(x\right)\right)}^{2}.$$

In this formula, *N* is the number of pixel pairs selected, while $x_{i}$ and $y_{i}$ stand for the values of the $i^{\mathrm{th}}$ pixel pair. Besides, E(x) represents the estimation of the mathematical expectations of x, and D(x) is the variance of x. Furthermore, cov(x,y) returns the estimation of the covariance between x and y. The correlation of adjacent pixels was reduced significantly, according to Figure 8 and Table 1. Moreover, the performance of the proposed scheme is compared to related works in Table 2, which helps to evaluate its performance objectively. As can be seen, the performance of the decorrelation by the proposed method was similar to those of existing works.

#### 4.1.5. Differential Analysis

The number of pixels change rate (NPCR) and the unified average changing intensity (UACI) are the general indicators of cryptosystems in the assessment of the performance in resisting differential attacks. Their definitions could be expressed as:

(14) $$NPCR=\sum_{ij}D(i,j)/n\cdot 100\%$$

and:

(15) $$UACI=\sum_{ij}abs\left(C1\left(i,j\right)-C2\left(i,j\right)\right)/\left(n\cdot 255\right)\cdot 100\%,$$

respectively. Here, abs(·) is the absolute value, and *n* denotes the number of pixels. In addition, D(i,j) and C1(i,j) represent the coherence of two cipher images in the location (i,j) and the pixel value of element (i,j) in Cipher Image 1, respectively. Specifically, the optimum values of NPCR and UACI in eight-bit gray-scale images of size 256×256 are 99.61% and 33.46%, respectively [42]. This paper assumed two similar plain images with a difference of one bit encrypted to be cipher C1 and cipher C2. Then, UACI could be obtained by Equation (15). Besides, the corresponding matrix D should be calculated to get the result of NPCR. To be specific, D(i,j) was set to zero as long as C1(i,j) was identical to C2(i,j). Otherwise, it should be set to one.

The results of NPCR and UACI with the proposed scheme are listed in Table 3. As this table shows, they were very close to the optimum values. Furthermore, the results of other works are compared with ours in Table 4. It can be found that the NPCR and UACI of the proposed encryption scheme were acceptable, but worse than some current methods. The primary reason was that, CBC mode was discarded by this paper for the purpose of boosting anti-jamming performance. In fact, if there exists a higher demand for NPCR and UACI, designers could manage this by adding more rounds of confusion and substitution operations.

#### 4.1.6. Randomness Analysis

Evaluating the randomness of cryptographic sequences generated by chaotic systems is necessary in designing encryption schemes. At present, most studies [43,44] utilized NIST SP 800-22 as their testing suite. It is a random number testing software approved by the National Institute of Standards (NIST). According to its requirements, the testing results’ *p*-values should be greater than the significance level α (α=0.01) if passing the corresponding tests. In this section, we construct a sample with a length of 4M bits by random parameters and initial states for implementing all 17 tests and list the results in Table 5. Obviously, the proposed OLM could successfully pass NIST SP 800-22 tests.

#### 4.1.7. Efficiency Analysis

Considering the parallel computing ability in the measurement process of CS, FPGA are preferred to be utilized as embedded processors in the front nodes. Although the hardware implementation has not been completed yet, theory analysis and simulation experiments can also evaluate the efficiency of the proposed scheme to some extent. According to the structure mentioned in Section 3, the proposed scheme merely added an operation of non-linear transform, in contrast to existing methods based on CS. Therefore, the time and space complexity of the designed method was still $O(CR\times L_{C})$, where $L_{C}$ is the number of pixels in a cipher image. The time consumption of the encryption and decryption process for the proposed method is listed in Table 6 in seconds. Besides, the results of some related references are compared in Table 7. Simulation results indicated that the efficiency of our scheme was high enough and approximate to other schemes. It is worth mentioning that the simulation experiments were performed on a Apple Laptop MD760 with the operating system Windows 7. In addition, MATLAB R2014b and plain images of size 256×256 were utilized.

#### 4.1.8. Information Entropy

Information entropy H(s) is taken as a statistical measure of uncertainty in communication theory [47]. It could be expressed by:

$$H\left(s\right)=\sum_{i=0}^{255}P\left(s_{i}\right)\log_{2}\frac{1}{P(s_{i})},$$

where *s* is a discrete random-variable and $P(s_{i})$ is the probability density function of the occurrence of $s_{i}$. If every symbol has an equal probability ($P\left(s_{i}\right)=1/2^{8},i=0,1,...,255$), then the entropy H(s)=8, and this is the ideal case. In the cryptosystem for images, the entropy of cipher images should be approximately eight. Simulation experiments were implemented, and the corresponding results are listed in Table 8. It can be found that the information entropy in cipher images was very close to the ideal value, and this means the encryption scheme can resist entropy attack.

### 4.2. Anti-Jamming Performance

When calculating the CR and the peak signal-to-noise ratio (PSNR) of a recovered image, most people might neglect the fact that all image data have finite precision. Since all pixel values were assumed to be UINT8, the formula of CR could be simplified from Equation (2) to Equation (1) under this hypothesis. Besides, our paper calculates the PSNR of the reconstructed image in the traditional way by:

$$PSNR=20\log_{10}\left(\frac{MAX_{I}}{\sqrt[{}]{MSE}}\right),$$

where $MAX_{I}$ is the maximum value of elements in an image ($MAX_{I}=255$ in this paper) and MSE is the mean squared error of the recovered image.

**Figure 9:**
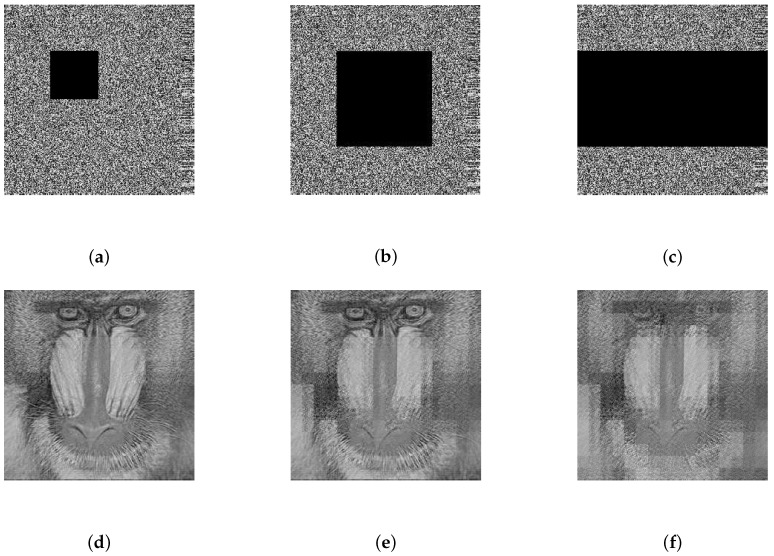
The cipher image of a baboon transmitted in the channel containing data loss of (**a**) 6.25%, (**b**) 25%, or (**c**) 50% and the corresponding decrypted image of the baboon containing data loss of (**d**) 6.25%, (**e**) 25%, or (**f**) 50%.

**Figure 10:**
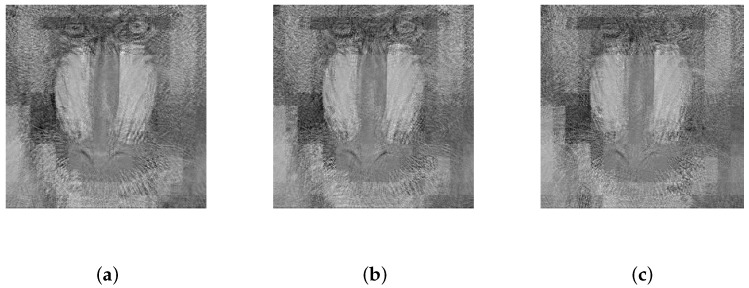
The decrypted image of a baboon transmitted by the channel containing AWGN with zero-mean and a standard deviation of (**a**) 40, (**b**) 60, or (**c**) 80.

According to Equation (12), the encryption model proposed depended on the statistical information of the plain image. Therefore, it was not sensitive to each element of the cipher image. As a result, the proposed scheme was more robust than the methods based on Equation (10) if the cipher image was altered. Since strong noise and severe data loss are major forms of interference in the wireless channel, this section simulates these situations to evaluate the anti-jamming performance of the proposed scheme. The image of the baboon was taken as an example to simulate 6.25%, 25%, or 50% occlusion in the corresponding cipher image, and it was also taken as an example to simulate zero-mean additive white Gaussian noise (AWGN) with standard deviations of 40, 60, or 80 in the corresponding cipher image. The results shown in Figure 9a–f and Figure 10a–c indicate that the proposed cryptosystem had satisfactory robustness.

**Figure 11:**
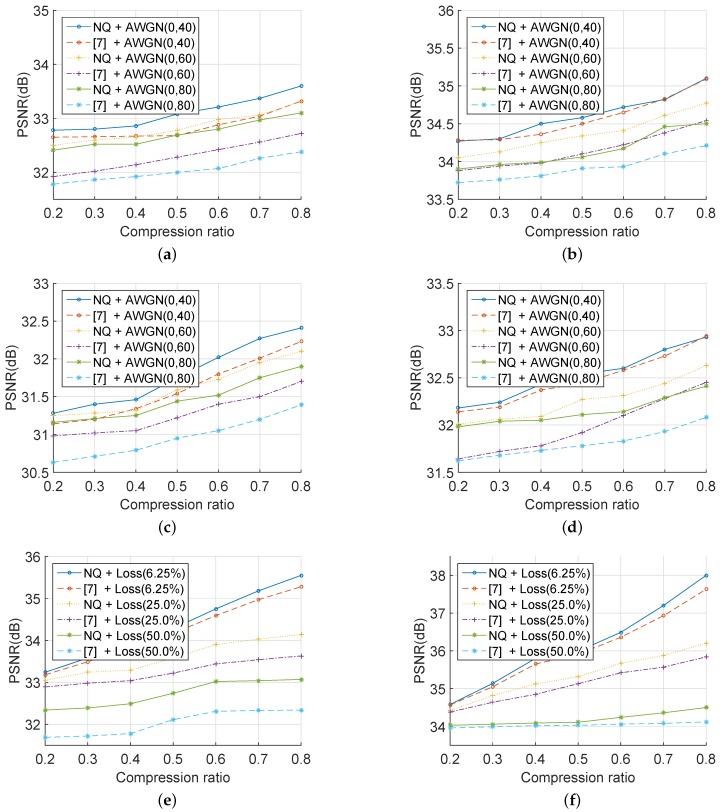

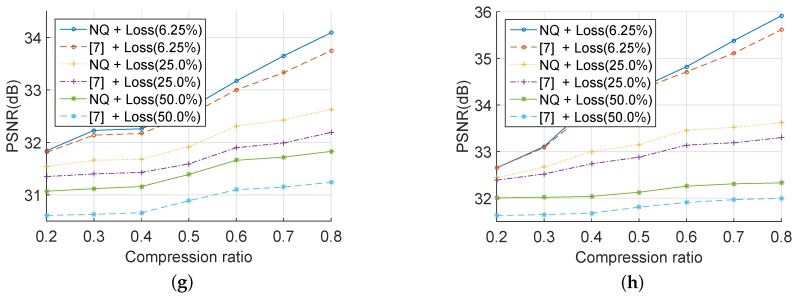
PSNR of decrypted images by the proposed scheme or [7] in different situations. AWGN with zero-mean and with a standard deviation of 40, 60, or 80 in the channel for the images: (**a**) baboon, (**b**) couple, (**c**) bridge, and (**d**) Lena; the data loss is 1/16, 1/4, or 1/2 in the channel for: (**e**) baboon, (**f**) couple, (**g**) bridge, and (**h**) Lena.

Then, with the same reconstruction algorithm and measurement matrix, the evaluation results were compared with those in [7]. Figure 11 illustrates that the PSNR of a recovered image was positively related to the CR of the corresponding cipher image. In other words, the outcome depended on the number of rows in the measurement matrix Φ. As can be seen, Figure 11a–d demonstrates that, under different compression ratios, the PSNR of recovered images with the proposed scheme was approximate to or higher than that with the method in [7] under powerful AWGN. Besides, Figure 11e–h shows that, in the situation of severe data loss, the PSNR of the decrypted images with the proposed scheme was better than that with the method in [7]. All results in Figure 11 were obtained by 100 Monte Carlo simulation experiments in MATLAB R2014b. In conclusion, the proposed scheme could help to improve the performance in adverse communication circumstances, such as in an intense AWGN environment or in a severe data loss situation.

## 5. Conclusions

To improve the security and anti-jamming performance in WVSN, this paper designed a secure and efficient cryptosystem with CS and NQ. In the proposed scheme, OLM expanded the parameter value space and eliminated the period windows in current chaotic systems. Besides, the cryptosystem proposed proved to have compression capability in a modified formula containing data precision. Furthermore, the utilization of NQ ensured that the encryption framework based on CS could resist CPA and retain its ability of anti-interference, which could not be realized in current research. However, the cryptosystem designed in this paper had some limitations that should be addressed in future work. Considering the lack of hardware implementation work and simple division for color images according to RGB, the most urgent needs for improvement are the details of the hardware implementation and the system optimization for color images. In short, the theories’ analysis and simulation results showed that the proposed scheme could resist CPA effectively. Furthermore, the original data could be efficiently compressed, and the performance of reconstruction in a harsh communication environment was improved. 

## Figures and Tables

**Table 1:** Correlation coefficients of adjacent pixel pairs in sample images for the proposed scheme.

	Vertical	Horizontal	Diagonal
Baboon/Cipher Baboon	0.7596/0.0013	0.8190/0.0006	0.7056/0.0042
Couple/Cipher Couple	0.9559/0.0057	0.9359/0.0075	0.9056/0.0043
Bridge/Cipher Bridge	0.8861/0.0073	0.9079/0.0129	0.8416/0.0072
Lena/Cipher Lena	0.9705/0.0089	0.9426/0.0125	0.9178/0.0006
Pepper/Cipher Pepper	0.9603/0.0024	0.9540/0.0309	0.9217/0.0059
Sailboat/Cipher Sailboat	0.9319/0.0050	0.9368/0.0353	0.8952/0.0025
Average of Plane/Cipher	0.9107/0.0050	0.9160/0.0166	0.8646/0.0041

**Table 2:** Correlation coefficients of adjacent pixel pairs in the encrypted Lena image for different cryptosystems.

	Vertical	Horizontal	Diagonal
Proposed scheme	0.0089	0.0125	0.0006
[38]	0.0006	0.0013	0.0019
[39]	0.0190	0.0127	0.0012
[40]	0.0015	0.0002	0.0040
[41]	0.0054	0.0045	0.0031

**Table 3:** NPCR and UACI of sample images for the proposed system.

	NPCR (%)	UACI (%)
Baboon	98.25	19.51
Couple	99.22	31.95
Bridge	98.93	23.34
Lena	98.84	24.03
Pepper	98.83	23.23
Sailboat	99.50	33.31
Average	98.93	25.90

**Table 4:** NPCR and UACI for existing cryptosystems.

	NPCR (%)	UACI (%)
Proposed scheme	98.93	25.90
[38]	99.61	33.45
[39]	99.65	33.48
[40]	99.63	33.39
[41]	99.61	33.38

**Table 5:** Randomness tests by NIST SP 800-22.

Test Name		*p*-Value	Conclusion
Approximate Entropy		0.643855	Pass
Block Frequency		0.559242	Pass
Cumulative Sums (Forward)		0.889834	Pass
Cumulative Sums (Reverse)		0.867819	Pass
FFT		0.229310	Pass
Frequency		0.977662	Pass
Linear Complexity		0.625158	Pass
Longest Runs of Ones		0.997956	Pass
Nonperiodic Templates		0.983870	Pass
Overlapping Template of All Ones		0.430142	Pass
Random Excursions	x=-4	0.418539	Pass
	x=-3	0.317595	Pass
	x=-2	0.722055	Pass
	x=-1	0.107461	Pass
	x=1	0.285961	Pass
	x=2	0.840700	Pass
	x=3	0.291290	Pass
	x=4	0.167648	Pass
Random Excursions Variant	x=-9	0.430201	Pass
	x=-8	0.320963	Pass
	x=-7	0.189480	Pass
	x=-6	0.138033	Pass
	x=-5	0.121120	Pass
	x=-4	0.183213	Pass
	x=-3	0.456083	Pass
	x=-2	0.709549	Pass
	x=-1	0.333201	Pass
	x=1	0.258912	Pass
	x=2	0.232093	Pass
	x=3	0.145987	Pass
	x=4	0.131251	Pass
	x=5	0.149733	Pass
	x=6	0.165778	Pass
	x=7	0.469584	Pass
	x=8	0.514143	Pass
	x=9	0.449499	Pass
Rank		0.549642	Pass
Runs		0.730847	Pass
Serial	*p*-value${}_{1}$	0.785632	Pass
	*p*-value${}_{2}$	0.711362	Pass
Universal Statistic		0.863189	Pass

**Table 6:** Time consumption of the encryption and decryption process for the proposed method.

	Encryption (s)	Decryption (s)
Baboon	0.17	1.5
Couple	0.16	1.5
Bridge	0.17	1.5
Lena	0.17	1.6
Mean	0.17	1.5

**Table 7:** Time consumption of the encryption and decryption process for the related references.

	Encryption (s)	Decryption (s)
Proposed scheme	0.17	1.5
[45]	0.23	0.21
[46]	1.5	2.0

**Table 8:** Information entropy of plain images and corresponding cipher images.

	Plain Image	Cipher Image
Baboon	7.1352	7.9966
Couple	6.3990	7.9967
Bridge	7.7282	7.9966
Lena	7.4962	7.9959
Mean	7.1897	7.9965

## Abbreviations

The following abbreviations are used in this manuscript:

WVSN	Wireless visual sensor networks
CS	Compressive sensing
CPA	Chosen-plaintext attack
NQ	Non-uniform quantization
CR	Compression ratio
LM	Logistic map
OLM	Optimized logistic map
CCA	Chosen-ciphertext attack
CBC	Cipher block chaining
UQ	Uniform quantization
RIP	Restricted isometry property
OMP	Orthogonal matching pursuit
ROMP	Regularized orthogonal matching pursuit
CCD	Charge coupled device
UINT8	Unsigned integers in eight bits
DCT	Discrete cosine transformation
XOR	Exclusive or
NPCR	The number of pixels change rate
UACI	The unified average changing intensity
PSNR	Peak signal-to-noise ratio
AWGN	Additive white Gaussian noise
